# Complex elbow dislocation associated with radial and ulnar diaphyseal fractures: a rare combination

**DOI:** 10.1007/s11751-011-0112-5

**Published:** 2011-07-20

**Authors:** Subramaniam Ramesh, Yi-Jia Lim

**Affiliations:** 1 Department of Orthopaedic Surgery, Tan Tock Seng Hospital, 11, Jalan Tan Tock Seng, Singapore, 308433 Singapore; 2 Department of Orthopaedic Surgery, Changi General Hospital, 2 Simei Street 3, Singapore, 529889 Singapore

**Keywords:** Elbow dislocation, Monteggia fracture, Radius, Ulna, Medial collateral ligament

## Abstract

We illustrate a rare complex dislocation of the elbow involving a posterior ulno-humeral dislocation associated with open diaphyseal fracture of the ulna, radial shaft fracture, Type 1 coronoid fracture and neuropraxia of the deep branch of the radial nerve. The isolated ulno-humeral dislocation without radio-capitellar involvement, and ulnar diaphyseal fracture, makes this “reverse Monteggia” type of injury pattern very unique. This patient was managed with an initial reduction of his ulno-humeral joint and stabilization of his radius and ulna fractures. He underwent a delayed medial collateral ligament reconstruction a few days later. His fractures went on to unite fully, his elbow joint remained stable, and he achieved good range of motion of his elbow.

## Introduction

Elbow dislocations are commonly associated with fractures around the elbow joint such as epicondylar fractures, radial head fractures or coronoid fractures [1]. The “terrible triad” of the elbow, consisting of a posterior dislocation together with a fracture of the coronoid process and the radial head, has been described before [2]. Ulnar diaphyseal forearm fracture associated with radial head dislocation has also been described before as the Monteggia fracture-dislocation [3]. Fracture-dislocations around the elbow usually involve the proximal radius or ulna [4]. However, elbow dislocations with concurrent fractures of both the ipsilateral radius and ulnar diaphyses are rare injuries. A few case reports have been published on this particular type of injury [5].

We illustrate a rare complex dislocation of the elbow involving a posterior ulno-humeral dislocation associated with open diaphyseal fracture of the ulna, radial shaft fracture, Type 1 coronoid fracture and nerve palsy of the deep branch of the radial nerve. The isolated ulno-humeral dislocation without radio-capitellar involvement makes this injury pattern very unique.

## Case report

A 20-year-old East Asian gentleman fell while playing *sepak takraw* (a local sport of kick volleyball native to Southeast Asia, resembling volleyball, except that it uses a rattan ball and only allows players to use their feet, knees, chest and head to touch the ball). He landed on his left arm after attempting an overhead kick. He subsequently complained of pain and deformity of his left arm with an open bleeding wound over the forearm.

He was assessed in an emergency department, and clinical examination revealed normal vital signs. His left elbow and forearm were deformed and swollen. There was a 5 mm bleeding puncture wound on the ulnar aspect (Gustilo Type 1) [6]. Radial and ulnar pulses were palpable. Careful examination of his distal motor and sensory function revealed finger drop suggestive of deep branch of radial nerve palsy. Sensation of the hand was intact. Examination of the elbow showed bruising and tenderness of the medial aspect. There was no tenderness or bruising over the radial aspect of the elbow joint.

**Fig. 1:**
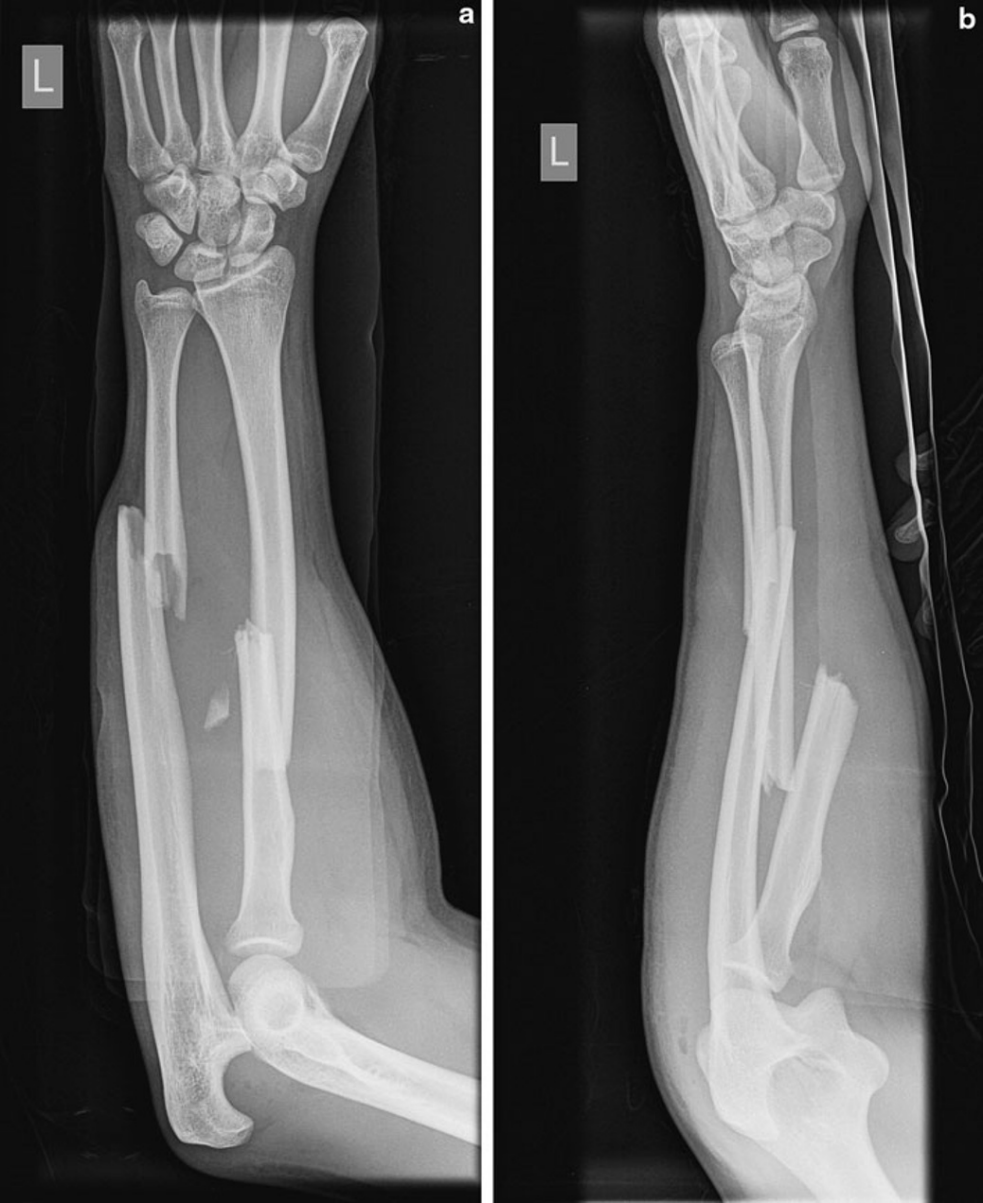
**a**, **b** Preoperative anteroposterior and lateral radiographs showing radius and ulna fractures with isolated ulno-humeral posterior dislocation

Radiographs revealed left radius and ulnar shaft fracture with posterior elbow dislocation and a Regan and Morrey Type 1 coronoid fracture. The radio-capitellar joint was enlocated (Fig. 1a, b).

He was given anti-tetanus and antibiotics (cefazolin and gentamicin) prophylaxis. Surgical intervention was recommended in view of the displaced fractures and joint dislocation.

### Operative intervention

He was brought to the operating theater, and his elbow joint was reduced with traction and manipulation. Reduction and joint congruity were confirmed with intraoperative fluoroscopy. The elbow was extremely swollen, and his medial collateral ligament was lax. This was demonstrated by opening up of the medial ulno-humeral joint space with valgus stress test under image intensifier.

He then underwent wound debridement, open reduction and internal fixation of his left radius and ulna. The ulnar-sided puncture wound was debrided and washed with copious amounts of saline. The bone ends of the fractured ulna were also irrigated. The radius fracture was fixed first due to the radial nerve palsy.

The skin incision was made, and the radius fracture was accessed using Henry’s approach. The radial nerve was noted to be intact and in continuity. There was comminution of the radius fracture with a medial butterfly fragment. The radius fracture was surgically stabilized using a 3.5 mm low contact dynamic compression plate (LC-DCP). The ulnar-sided open wound was then extended along the subcutaneous border of the ulna. The ulna fracture was stabilized using a 3.5 mm LC-DCP as well.

The patient underwent examination under general anaesthesia 4 days later when the elbow swelling was less. There was medial laxity noted on valgus stress in 30° of flexion. There was no posterolateral instability, and re-dislocation of the elbow was not possible. We proceeded to repair his medial collateral ligament. This was done via an arcuate incision made along the medial epicondyle. Intraoperatively, the medial collateral ligament was noted to be totally avulsed from its proximal insertion on the medial epicondyle. The medial collateral ligament was reattached to its proximal insertion using a 3 mm Mitek (DePuy, Johnson and Johnson) suture anchor. No exploration of the lateral ligaments was performed as there was no lateral laxity or bruising, and the radial head was enlocated. Intraoperative assessment of the elbow joint after repair of the medial collateral ligament showed stability on valgus stress and no posterolateral instability. The ulnar nerve was also noted to be intact. As there was also no further anteroposterior instability, the Type I coronoid fracture was left alone. His elbow was further immobilized for a week in a backslab.

His postoperative recovery was uneventful, and he completed 4 days of intravenous antibiotics. He was discharged home on the fifth postoperative day with oral antibiotics (cephalexin) for a further 5 days. Thereafter, he was followed up in the specialist outpatient clinic. The patient was not treated with nonsteroidal anti-inflammatories (NSAIDs) or radiotherapy, as he had no other risk factors for heterotrophic ossification. He was started on early progressive range of motion exercises to prevent elbow stiffness.

At 5 months after surgery, his range of motion of his left elbow was 0°–135°. Pronation and supination was 70° each. Valgus stress test and test for posterolateral instability were negative. His wounds had healed well. His finger drop resolved, and he was able to move his fingers completely. His grip and pinch strength had also normalized by comparison to the contralateral hand.

**Fig. 2:**
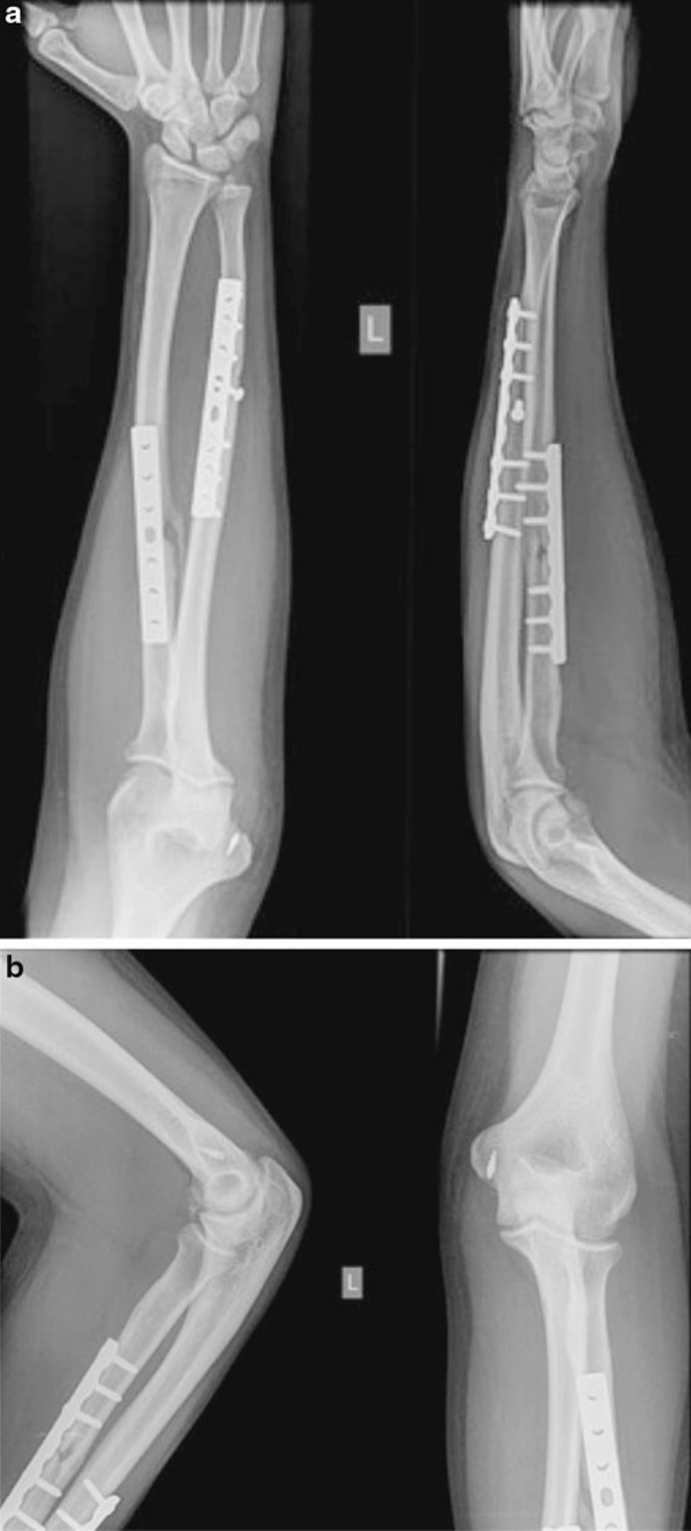
**a**, **b** Radiographs of the left elbow and forearm 5 months after the surgery

Xrays revealed bony union, and his elbow joint was congruent. He was encouraged to continue his physiotherapy to improve his range of motion (Fig. 2a, b).

## Discussion

The elbow joint is one of the most inherently stable articulations of the human skeletal system. The hinged ulno-humeral articulation, the radio-humeral articulation and the proximal radio-ulnar articulation together with the ligamentous stability conferred by the medial collateral ligaments and lateral collateral ligaments lend to its remarkable stability [7]. There are also secondary constraints that contribute to varus and valgus stability, including the radial head, the common flexor and extensor origins and the capsule of the elbow joint. The term “terrible triad” was coined by Hotchkiss [2] to describe the combination of elbow dislocation, radial head and coronoid process fractures which often leads to major disability and complications such as persistent instability, nonunion, malunion and proximal radio-ulnar osseous synostosis.

While the terrible triad combination of elbow injuries is commonly seen, it is rare for elbow dislocation to be associated with diaphyseal radius and ulna fractures. In 1814, Giovanni Monteggia described two cases of fracture of the proximal ulna with anterior dislocation of the radial head. Bado classified the Monteggia lesion into four different types. The Bado Type IV lesion classifies ipsilateral fractures of the radius and ulnar shaft with radial head anterior dislocation. However, none of the classifications take into account proximal ulnar shaft fracture and radial shaft fracture, with concomitant elbow dislocation involving the ulno-humeral joint.

This rare injury of radius and ulnar shaft fractures with elbow posterior dislocation has been described as a unique Monteggia-equivalent injury by Hung et al. [5] and Kose et al. [8]. The postulated mechanism of injury in these injuries is the initial elbow dislocation as a result of the fall on the outstretched arm. Then, both the bones of the forearm fractured while the elbow was in extension, the forearm in hyper-pronation and the wrist in radial deviation. It is unlikely for the elbow dislocation to follow fractures of both the forearm bones.

Coronoid process fractures have been identified in 5–10% of elbow dislocations [9, 10]. They rarely occur in isolation and are usually associated with radial head fractures, elbow dislocations or both(terrible triad of elbow) [11]. Closkey et al. [12] in their biomechanical studies showed that if more than 50% of the coronoid process was fractured, the elbow tends to displace more readily in response to axial loads. Regan and Morrey also showed that the amount of coronoid process that is fractured directly relates to the prognosis and prevalence of instability [9]. The elbow in full extension with axial load from the patient’s weight probably led to his coronoid process fracture in the process of his elbow dislocation. The medial extension of the coronoid fracture also has implications in the medial stability of the elbow as it bears the attachment of the anterior bundle of the medial collateral ligament [13].

Palsy of the deep branch of the radial nerve is most common in Bado Type II Monteggia fractures [14]. Posterior displacement of the radial head causes deep branch of the radial nerve palsy at the time of injury and dislocation or as a delayed presentation due to constant pressure. The proximity of the nerve to the radial head and its course through the arcade of Frohse makes it susceptible to injury, from the initial traumatic event or the resultant edema. Diaphyseal fractures of the radius and ulna have also been shown to cause traumatic injuries of the nerve. Fortunately, nerve palsy in these injuries is mainly due to neuropraxia of this nerve, which often resolves with conservative management, as was the case with our patient. Since there was no lateral elbow bruising or lateral ligamentous laxity, and the initial Xrays did not reveal a radio-capitellar dislocation, we postulate that the palsy of the deep branch of the radial nerve was most likely due to the diaphyseal fractures or edema from the complex dislocation.

This case illustrates a rare combination of joint dislocation (elbow), bony fractures (radius, ulnar shaft and coronoid process), nerve injury (deep branch of radial nerve palsy) and ligamentous disruption (medial collateral ligament). Though Hung et al. [5] had previously described a unique Monteggia-equivalent elbow injury, we report a very unique complex elbow dislocation that has resulted in isolated ulno-humeral dislocation without involvement of the radio-capitellar joint. This pattern of injury may still be a Monteggia equivalent due to the ulnar diaphyseal fracture and proximal joint dislocation. We feel it may be more accurate to describe this configuration of injury as a “reverse Monteggia” type of fracture-dislocation where the ulnar diaphyseal fracture is not associated with a radio-capitellar dislocation, but an isolated ulno-humeral dislocation instead.

This explains the injuries to the medial collateral ligament and coronoid process, while the lateral ulnar collateral ligament and radial head were both intact in this situation, providing some stability to the elbow after initial reduction. The medial collateral ligament was repaired as intraoperative assessment of the elbow-revealed valgus laxity as evidenced by the opening up of the medial ulno-humeral joint space with valgus stress test under image intensifier.

There is little evidence in the literature that operative repair of the collateral ligaments has significant advantage over nonsurgical treatment in a plaster cast [15]. For surgical treatment of the collateral ligaments, medial collateral ligament reconstruction is particularly indicated when there is residual instability following lateral ligament reconstruction or in throwing athletes [16, 17]. We chose to repair the medial collateral ligament in our patient, as we did not want prolonged immobilization for ligament healing, and to start early range of motion exercises. There has also been recent evidence to suggest good results with acute repair of the medial collateral ligament [18]. The use of a hinged external fixator would also have been an option to allow early range of motion exercises and protect the collaterals [19].

In managing such a complex elbow fracture-dislocation, there must be due consideration for restoration of joint congruity, skeletal stabilization as well as ligamentous reconstruction with careful handling of soft tissues around the elbow joint. As such, a combined single stage approach for radius and ulnar shaft stabilization with joint reduction and collateral ligament reconstruction may be useful. However, this complex injury resulted in significant soft tissue edema, and we had elected to reduce the joint and achieve skeletal stabilization for the diaphyseal fractures, followed by delayed surgery for his elbow ligamentous reconstruction once the edema had subsided.
